# NAND and NOR logic-in-memory comprising silicon nanowire feedback field-effect transistors

**DOI:** 10.1038/s41598-022-07368-0

**Published:** 2022-03-07

**Authors:** Yejin Yang, Juhee Jeon, Jaemin Son, Kyoungah Cho, Sangsig Kim

**Affiliations:** 1 Department of Semiconductor Systems Engineering, Korea University, Seoul, Republic of Korea; 2 Department of Electrical Engineering, Korea University, 145 Anam-ro, Seongbuk-gu, Seoul, 02841 Republic of Korea

**Keywords:** Engineering, Electrical and electronic engineering

## Abstract

The processing of large amounts of data requires a high energy efficiency and fast processing time for high-performance computing systems. However, conventional von Neumann computing systems have performance limitations because of bottlenecks in data movement between separated processing and memory hierarchy, which causes latency and high power consumption. To overcome this hindrance, logic-in-memory (LIM) has been proposed that performs both data processing and memory operations. Here, we present a NAND and NOR LIM composed of silicon nanowire feedback field-effect transistors, whose configuration resembles that of CMOS logic gate circuits. The LIM can perform memory operations to retain its output logic under zero-bias conditions as well as logic operations with a high processing speed of nanoseconds. The newly proposed dynamic voltage-transfer characteristics verify the operating principle of the LIM. This study demonstrates that the NAND and NOR LIM has promising potential to resolve power and processing speed issues.

## Introduction

As we have entered the era of the Internet of Things, Big Data, and artificial intelligence, the global need for data processing has exponentially increased in various fields such as medical services, industrial production, and social media^1–5^. Processing large amounts of data requires high-performance and energy-efficient computation, which is a critical factor for a wide range of data-intensive applications. However, current computer systems based on the von Neumann architecture incur significant power consumption and latency because of the speed gap when accessing data between the separated central processing and memory units^6–9^. Recently, logic-in-memory (LIM) has been investigated to overcome the architectural limitations of data movement. LIM performs logical operations in a memory unit to eliminate data movement between the logical and memory tasks. Therefore, LIM architecture has the advantages of not only high bandwidth memory, but also highly energy-efficient computing and processing time.

Various LIM devices can be implemented using either charge-based or resistance-based memory. Charge-based memory mainly refers to static random-access memory (SRAM), dynamic RAM (DRAM), and flash memory. Resistance-based memory includes resistive RAM (RRAM), spin-transfer torque magnetoresistive RAM (STT-MRAM), and phase-change memory (PCM). The read/write time, voltage, LIM computation energy/latency, leakage power, and retention for existing charge-or resistance-based memory, as reported in other studies, are listed in Table 1^10–14^. In charge-based memory, although SRAM is very fast (~ 1 ns) and DRAM has high density, they contain volatile memory devices, and hence have high energy needs. Moreover, SRAM-based LIM suffers from chip area overhead because more transistors than in the conventional 6 T SRAM cells are required for computing operations^15,16^. Likewise, DRAM-based LIM has the challenges of area and yield due to the limitation of the cell structure^16–18^. NAND/ NOR flash memory with a charge-trapping layer can store data long-term. Nevertheless, for LIM operation, it not only has a low read/write time (< 103/ < 106 ns) and a high operating voltage (< 10 V), but also a relatively high computation energy (41.62/ 0.2 nJ) and latency (8421/ ~ 500 ns).

**Table 1:** Comparison of our NAND and NOR LIM and existing LIM technologies^10–14^.

	Ref	SRAM	DRAM	NAND Flash/NOR Flash	RRAM	STT-MRAM	PCM	NAND LIM/NOR LIM
Read time (ns)	^11^	~ 1	~ 10	~ 10^4^/~ 50	< 10	< 10	< 10	< 5
Write time (ns)	^11^	~ 1	~ 10	10^5^_–_10^6^/10^4^_–_10^6^	< 10	< 5	~ 50	< 5
Voltage (V)	^11^	< 1	< 1	< 10	< 3	< 2	< 3	≤ 2.5
LIM computation energy (ns)	^10,13,14^	~ 0.59 nJ	~ 0.75 Nj	41.62 pJ/0.2 pJ	~ 1.13 nJ	~ 0.79 nJ	–	~ 0.5 pJ/0.2 pJ
LIM computation latency (ns)	^10,13,14^	3.1	13.6	8421/~ 500	~ 1.9	~ 3.48	–	~ 1
Leakage power	^12^	High	Medium	Low	Low	Low	Low	Low
Retention	^11^	N/A	~ 64 ms	> 10 y	> 10 y	> 10 y	> 10 y	> 10 s/> 26 s

New emerging resistance-based memory (RRAM, PCM, and STT-MRAM) has a non-volatile memory (NVM) capability, and LIM based on resistance-based memory has been presented^19–24^. However, resistance-based memory has several drawbacks, such as low processing speed, high operating voltage, and fabrication process. Specifically, RRAM-based LIM has inconsistent switching characteristics due to variations in the fabrication process and requires a relatively high voltage (< 3 V) to generate a high current compared to conventional volatile memories^24,25^. The PCM-based LIM has a low write speed (~ 50 ns) compared to other memory devices due to the switching between crystalline and amorphous phases, and a comparatively high operating voltage (< 3 V)^26^. The STT-MRAM-based LIM has a low chip yield for mass production, low reliability because of stochastic switching, and read disturbances^27^. Moreover, resistance-based memory requires new fabrication processes and new materials (not based on CMOS fabrication technology), and it is not yet mature enough to be available for commercial technology/products^18,28^.

In this study, we propose a NAND and NOR LIM composed of silicon nanowire (SiNW) feedback field-effect transistors (FBFETs) to verify universal gate functions, where the configuration of the SiNW FBFETs maintains conventional CMOS logic gates. The SiNW FBFETs utilized in the LIM have demonstrated near-zero subthreshold swings (*SS*), high speed, low operating voltage, and quasi-nonvolatile memory characteristics based on the positive feedback loop mechanism^29–31^. The LIM exhibits a high processing speed close to that of SRAM and DRAM (< 5 ns), ultra-low standby power while storing the data, retention characteristics that will retain certain computational logic states without power supply, and relatively low operating voltage (≤ 2.5 V) compared to flash memory. Furthermore, the LIM exhibits a relatively high density compared with charge-based memory because it implements LIM operation on only four FBFETs without separate storage devices. The proposed LIM has excellent gate controllability of charge carriers in the silicon channel and has a simple fabrication process by CMOS-compatible top-down technology^32,33^. The FBFETs could be produced using commercially available fabrication processes of conventional GAA SiNWs, and our LIM could be implemented by arranging the FBFETs as CMOS logic circuit^31^. Thus, it is feasible that our LIM is made in commercially available fabrication processes^34^. Therefore, the presented LIM can not only reduce fabrication costs but also enable rapid commercialization in the LIM market. Moreover, the presented LIM has comparatively low LIM computation energy (~ 0.5/ ~ 0.2 pJ) and latency (~ 1 ns) compared with the figures-of-merit of LIM using other memory devices. Furthermore, we specifically examined the operations of the NAND and NOR LIM using the newly proposed dynamic voltage-transfer characteristics (VTC). We investigated the feasibility of the NAND and NOR LIM through mixed-mode technology computer-aided design (TCAD) simulations^35^.

### Schematic of the FBFETs and circuit diagrams of the LIM

**Figure 1:**
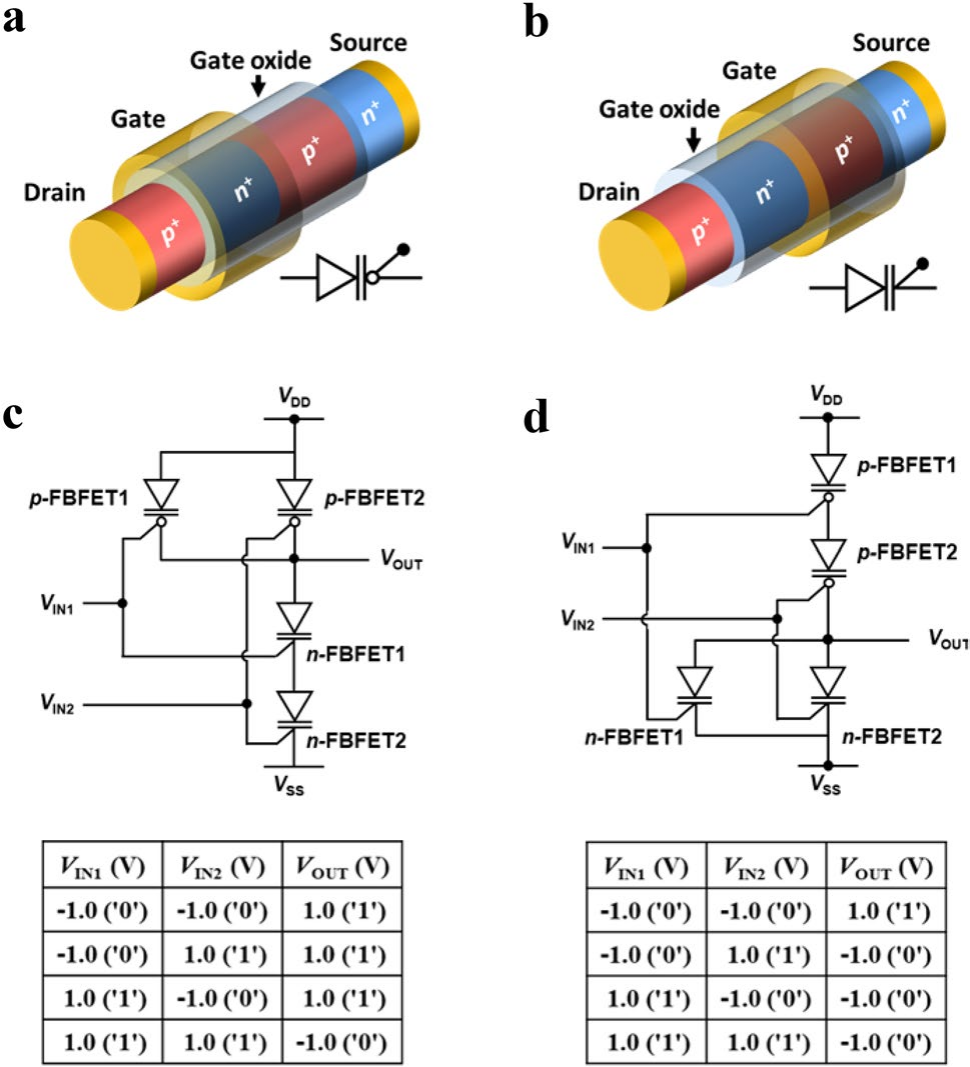
Structure of SiNW *p*- and *n*-FBFETs and diagrams of LIM. (**a**,**b**) Schematic view of single-gated SiNW (**a**) *p*- and (**b**) *n*-FBFETs. (**c**,**d**) Diagrams and truth tables of (**c**) NAND and (**d**) NOR LIM circuits consisting of *p*- and *n*-FBFETs.

Figure 1 shows a schematic view of a single-gated SiNW *p*-channel FBFET (*p*-FBFET) and a single-gated SiNW *n*-channel FBFET (*n*-FBFET) utilized in the NAND and NOR LIM. The dimensional parameters of the *p*-FBFET, as shown in Fig. 1a, are a channel length (*L*_CH_) of 150 nm, *p*^+^ drain and *n*^+^ source region lengths of 50 nm, and a silicon channel diameter of 25 nm. The dimensional parameters of the *n*-FBFET, as shown in Fig. 1b, are an *L*_CH_ of 200 nm, *p*^+^ drain, and *n*^+^ source region lengths of 50 nm, and a silicon channel diameter of 15 nm. The non-gated and gated channel lengths are 1/2*L*_CH_ for both FBFETs. Supplementary Section 1 provides more details on the device dimensional parameters. Figure 1c,d show the diagrams and truth tables of the NAND and NOR LIM, respectively, consisting of two *n*-FBFETs, two *p*-FBFETs, and a parasitic load capacitor connected to the output node with a capacitance of 10 fF^36,37^. The parasitic load capacitance represents the capacitance of the wire or interconnect between the output of the logic gate and the input of another logic gate. These LIM operations are conducted by applying voltage pulses of supply voltages *V*_DD_ and *V*_SS_ (defined as the drain voltage of the *p*-FBFET and the source voltage of the *n*-FBFET, respectively), gate input voltage 1 (*V*_IN1_), and gate input voltage 2 (*V*_IN2_) under dynamic conditions.

## Results and discussion

### Operating principle of the FBFETs constituting the LIMs

The operation principle of the *p*- and *n*-FBFETs is based on a positive feedback loop mechanism in the channel regions^29–31^. The FBFETs consisting of *p*^+^–*n*^+^–*p*^+^–*n*^+^ regions have two potential barriers in the channel regions, and the potential barrier heights are controlled by the presence or absence of charge carriers in the potential well. For the off-state under a gate voltage (*V*_GS_) of 1 V (or − 1 V) and positive drain voltage (*V*_DS_) in the *p*-FBFET (or *n*-FBFET), the potential barrier near the drain side of the channel region blocks the injection of holes from the drain into the channel region. Likewise, the potential barrier near the source side blocks the injection of electrons from the source. During the *V*_GS_ negative (or positive) sweep, the charge carriers are injected into the channel regions and accumulate in the potential wells; the accumulated charge carriers lower the potential barrier height in the channel regions. When the repetition of injection and accumulation of charge carriers generates a positive feedback loop, the two potential barriers collapse abruptly, and the drain current (*I*_DS_) rapidly increases. Thus, the *p*-FBFET (or *n*-FBFET) changes to an on-state under a *V*_GS_ of − 1 V (or 1 V). During hold operation under no-bias conditions, the accumulated charge carriers in the potential wells are maintained until the potential barriers in the channels are well-defined, and the FBFETs have quasi-nonvolatile memory characteristics^38,39^. In the *V*_GS_ positive (or negative) sweep for the *p*-FBFET (or *n*-FBFET), the charge carriers accumulated in the channel regions are reduced, and the two potential barriers are regenerated. Consequently, the positive feedback loop is eliminated, and the *I*_DS_ rapidly decreases; thus, the *p*-FBFET (or *n*-FBFET) changes to an off-state.

### NAND and NOR LIM operation under dynamic conditions

**Figure 2:**
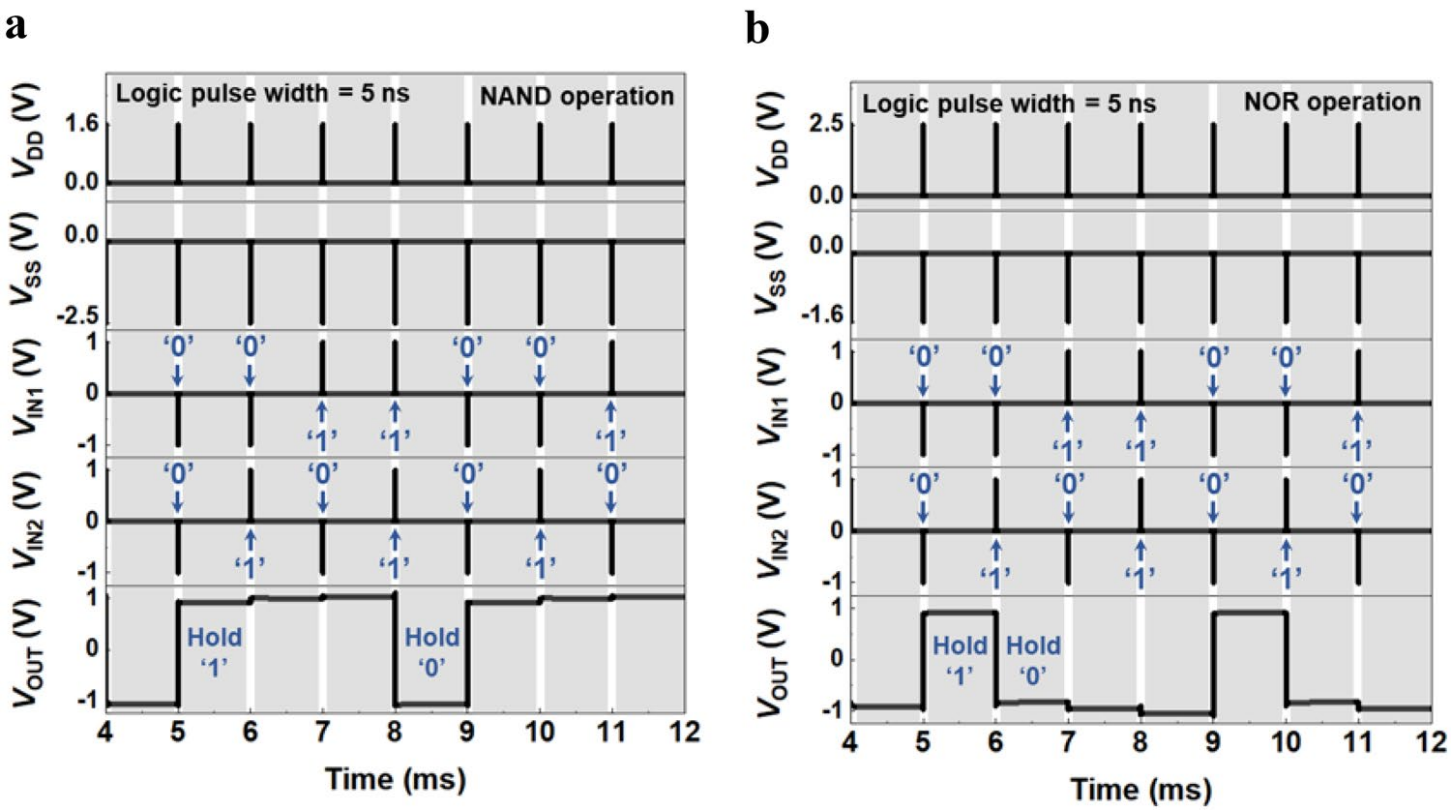
LIM operations. (**a**,**b**) Timing diagrams of the (**a**) NAND and (**b**) NOR LIM operations with input voltages (*V*_DD_, *V*_SS_, *V*_IN1_, and *V*_IN2_) and *V*_OUT_. Each logic pulse width is 5 ns and the hold time is 1 ms.

Figure 2a shows both the logic operation of the NAND LIM and the memory operation to hold its output logic states (for more details of the logic operation, see Supplementary Section 2). The time width of the input logic pulse is 5 ns, corresponding to a frequency of 0.2 GHz, and the hold time width is 1 ms, corresponding to a frequency of 1 kHz. In the timing diagram in this figure, the LIM operations are performed in a input logic gate combination sequence of ‘00’, ‘01’, ‘10’, and ‘11’, and after each logic operation, the memory operation to hold the output logic states is performed without any external bias (*V*_DD_ = *V*_SS_ = *V*_IN1_ = *V*_IN2_ = 0.0 V). First, the input logic ‘00’ of the NAND LIM operates with *V*_DD_ = 1.6 V, *V*_SS_ =  − 2.5 V, *V*_IN1_ =  − 1.0 V, and *V*_IN2_ =  − 1.0 V, providing the output logic ‘1’ (*V*_OUT_ = 1.0 V). The output logic ‘1’ is stored for 1 ms in the channel region of the FBFETs without any external bias supplies, and the operation is referred to as the hold ‘1’ operation. When the input logic ‘00’ with *V*_IN1_ = *V*_IN2_ =  − 1.0 V is applied, the two *p*-FBFETs are turned on, and the two *n*-FBFETs are simultaneously turned off. Thereafter, during the hold ‘1’ operation under zero-bias conditions, the output logic value is stored by the quasi-nonvolatile memory characteristics of the FBFETs^30,38,39^. The input logic ‘01’ operates with *V*_IN1_ =  − 1.0 V and *V*_IN2_ = 1.0 V, and the output logic ‘1’ is provided. The state in which the output logic ‘1’ is stored under zero-bias conditions is referred to as the hold ‘1’ operation. The input logic gate ‘10’ operates with *V*_IN1_ = 1.0 V and *V*_IN2_ =  − 1.0 V, and the output logic ‘1’ is provided, and thereby the output logic is stored without any voltages applied. The input logic gate ‘11’ operates with *V*_IN1_ = 1.0 V and *V*_IN2_ = 1.0 V, and the output logic transits from ‘1’ to ‘0’, which indicates *V*_OUT_ =  − 1.0 V because a current path is created between *V*_OUT_ and *V*_SS_. The output logic ‘0’ is also stored and maintained under zero-bias conditions corresponding to the hold ‘0’ operation. For the write operations, the calculated average write energy is approximately 0.5 pJ/bit. In addition, for the read operations, no sensing voltage is required to read the *V*_OUT_ because the logic states of the NAND LIM are stored as *V*_OUT_. The NAND and NOR LIMs are compared in Table 1 with existing charge- or resistance-based memory technologies reported by other research groups in terms of the read/write time, voltage, LIM computation energy/latency, leakage power, and retention^10–14^.

Figure 2b shows both the logic operation of the NOR LIM and the memory operation to hold its output logic states (for more details of the logic operation, see Supplementary Section 2). In the timing diagram, the LIM operations are carried out in an input logic gate combination sequence of ‘00’, ‘01’, ‘10’, and ‘11’, and after each logic operation, the memory operation to hold the output logic states is performed without any external bias. First, the input logic gate ‘00’ of the NOR LIM operates with *V*_DD_ = 2.5 V, *V*_SS_ =  − 1.6 V, *V*_IN1_ =  − 1.0 V, and *V*_IN2_ =  − 1.0 V, and the output logic ‘1’ is carried out. Thereafter, the output state is stored under zero-bias conditions, which is referred to as the hold ‘1’ operation. The input logic gate ‘01’ operates with *V*_IN1_ =  − 1.0 V and *V*_IN2_ = 1.0 V, and the output logic transits from ‘1’ to ‘0’. The output logic ‘0’ is also stored under zero-bias conditions while blocking the current path. The input logic gate ‘10’ operates with *V*_IN1_ = 1.0 V and *V*_IN2_ =  − 1.0 V, resulting in output logic ‘0’, and thereby the hold ‘0’ state is maintained. Subsequently, the input logic gate ‘11’ operates with *V*_IN1_ = 1.0 V and *V*_IN2_ = 1.0 V, and the output state is stored after the output logic ‘0’ is performed because two *n*-FBFETs are turned on, which pulls *V*_OUT_ down to *V*_SS_. For the write operations, the calculated average write energy is approximately 0.2 pJ/bit. In addition, for the read operations, no sensing voltage is required to read the *V*_OUT_ because the logic states of the NOR LIM are stored as *V*_OUT_.

**Figure 3:**
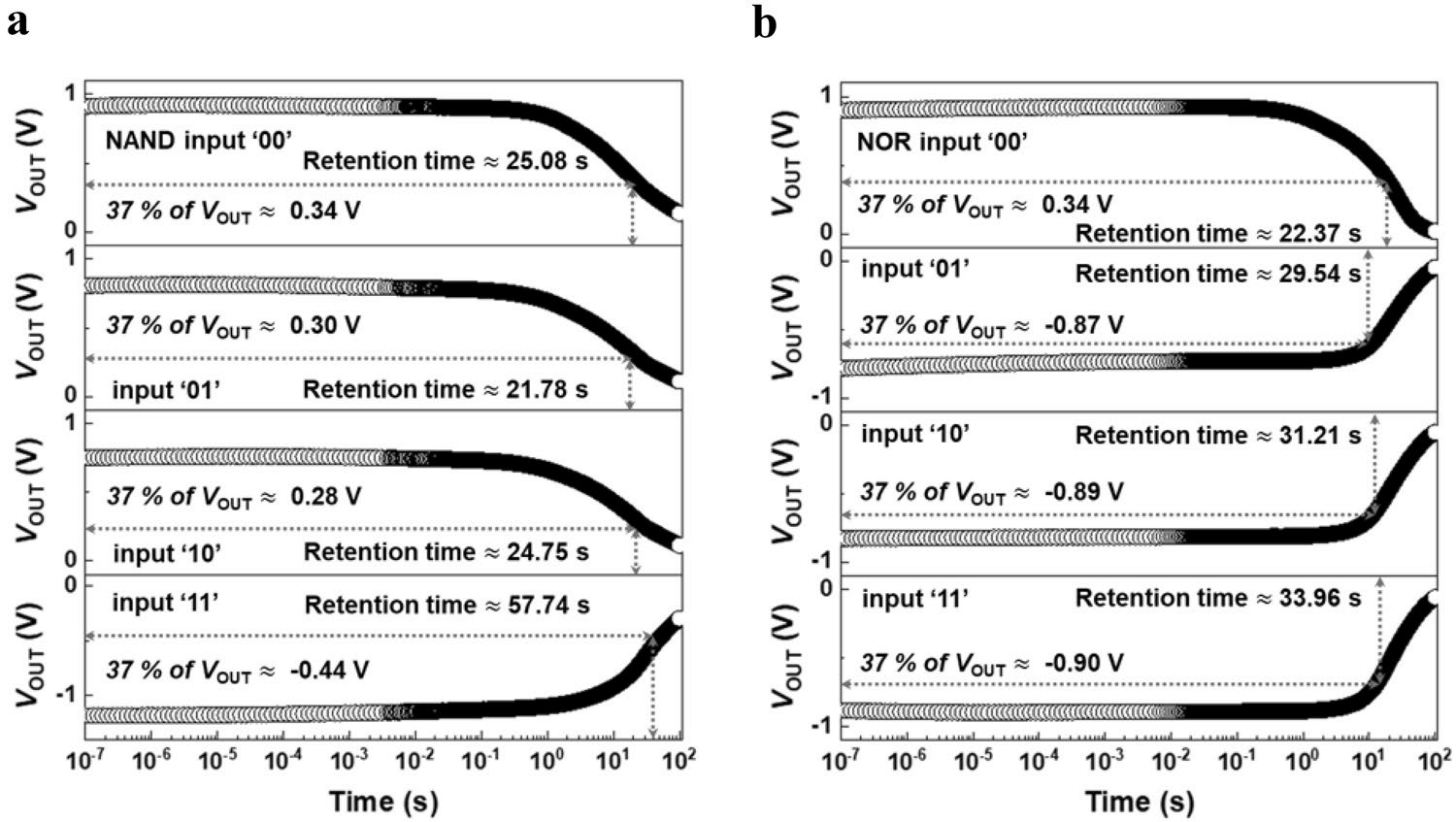
LIM retention characteristics. (**a**) Time-dependent retention characteristics for *V*_OUT_ of NAND LIM, following the input logic of ‘00’, ‘01’, ‘10’, and ‘11’ with each pulse width of 5 ns, respectively. (**b**) Time-dependent retention characteristics for *V*_OUT_ of NOR LIM, following the input logic of ‘00’, ‘01’, ‘10’, and ‘11’ with each pulse width of 5 ns, respectively. The retention time for output logic is the time corresponding to 37% of the initial *V*_OUT_.

To demonstrate the retention time of holding the output logic state after the input logic operation of the NAND and NOR LIM, we initialized the output logic to ‘0’. Subsequently, input voltages corresponding to the input logic with a pulse width of 5 ns were applied, and all zero-bias conditions were maintained for 100 s, as shown in Fig. 3. The retention time is calculated as the time at which the output voltage reaches 37% of the initial *V*_OUT._ As a result, the retention time of the NAND LIM corresponding to the input logic sequence ‘00’, ‘01’, ‘10’, and ‘11’ is 25.08 s, 21.78 s, 24.75 s, and 57.84 s, which represents 37% of the initial *V*_OUT_ as shown in Fig. 3a; note that the retention time of the presented LIM is compared in Table 1 with existing charge- or resistance-based memory. Likewise, the retention time of the NOR LIM corresponding to the input logic sequence ‘00’, ‘01’, ‘10’, and ‘11’ is 22.37 s, 29.54 s, 31.21 s, and 33.96 s as shown in Fig. 3b. The retention time of the NAND and NOR LIM is affected by the parasitic capacitor since the output value is temporarily stored in the parasitic capacitor after the dynamic logic operation; the retention time of the logic circuit including the parasitic capacitor is longer than that of the logic circuit without the parasitic capacitor. On the other hand, the logic circuit composed of MOSFETs with the parasitic capacitor does not maintain the logic operation values, in contradistinction to the logic circuit composed of FBFETs with the parasitic capacitor. Changes in retention time depending on the parasitic load capacitance are described in our supplementary section 3. Although the same output states of ‘0’ or’1’ are carried out in the NAND and NOR LIM, there is a slight difference in the retention times. This is because each of the FBFETs has a difference in on-/off-resistance due to a slightly different potential barrier formed in the channel region when the transient switching is operated by the four input pulses^40,41^. This leads to different resistance ratios between the pull-up and pull-down networks. On the other hand, the use of the different supply voltages becomes a bottleneck in a commercial circuit, and hence the matching of the resistances between the pull-up and -down networks is needed to overcome the supply voltage problem. The resistance ratio determines the *V*_OUT_ value by dividing the voltage of the supply voltages on each node of the NAND and NOR LIM. Thereafter, the *V*_OUT_ is retained without any external bias as long as the resistance ratio is maintained because of the presence of a potential barrier that modulates over time. Consequently, the retention characteristics depend not only on the retention feature of a single FBFET, but also on the circuit configuration where the supply voltages are divided.

### Dynamic voltage-transfer characteristics of the LIMs

**Figure 4:**
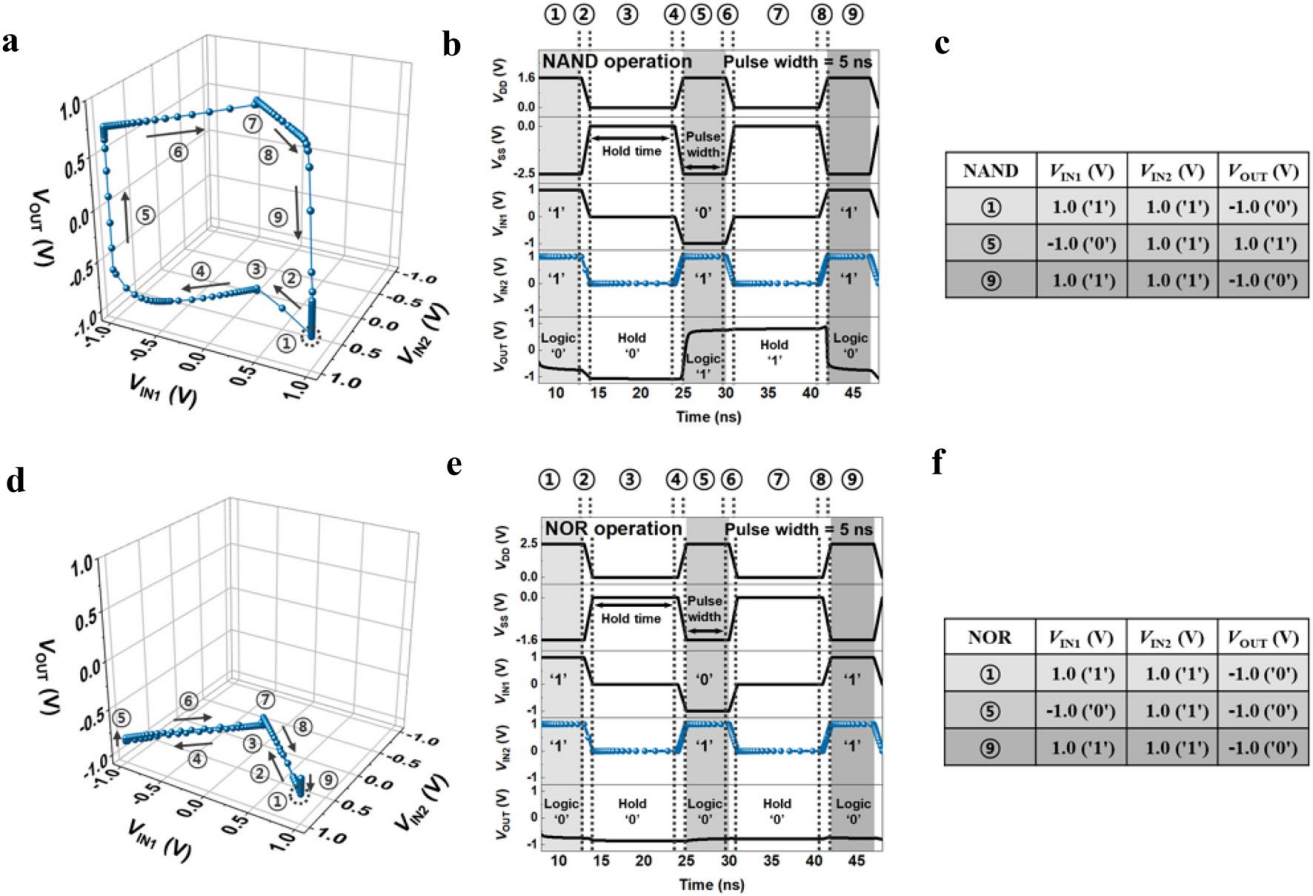
VTCs demonstration with a certain sequence. (**a**) Dynamic VTCs of the NAND LIM sweeping *V*_IN1_ with pulse values of (a) *V*_IN2_ = 1.0 V. (**b**) Timing diagrams corresponding to the sequential dynamic VTCs. (**c**) Tables (**c**) summarize the performed logic operations. (**d**) Dynamic VTC of the NOR LIM in a sweep of *V*_IN1_ with pulse values of (**d**) *V*_IN2_ = 1.0 V. **e**, Timing diagrams corresponding to the sequential dynamic VTCs. (**f**) Tables (**f**) summarize the performed logic operations.

**Figure 5:**
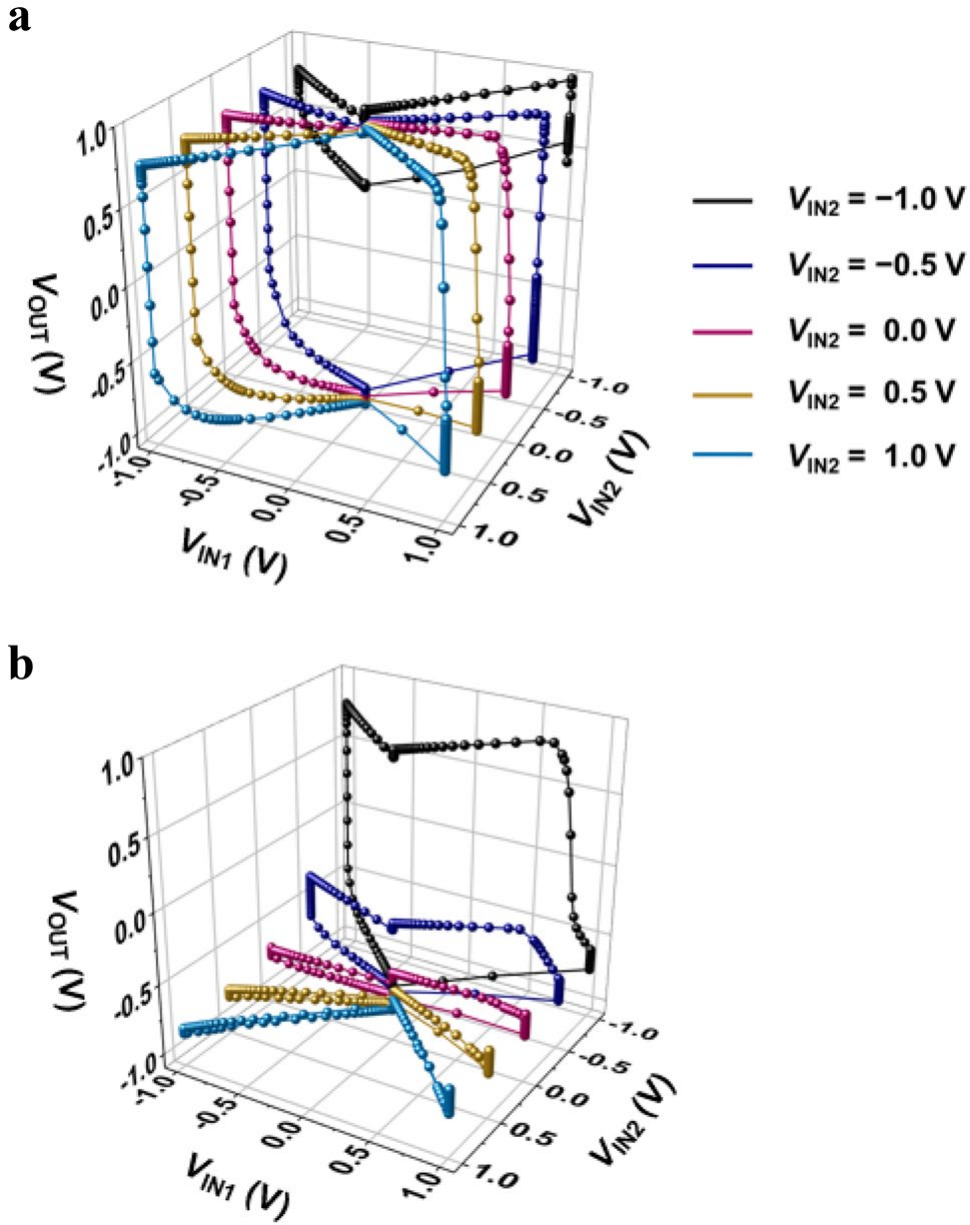
VTCs for the NAND and NOR LIM. (**a**) Dynamic VTCs of the NAND LIM sweeping *V*_IN1_ with different pulse values of *V*_IN2_. (**b**) Dynamic VTCs of the NOR LIM sweeping *V*_IN1_ with different pulse values of *V*_IN2_.

We newly defined the dynamic VTC of the NAND and NOR LIM for two input voltages to demonstrate the operation mechanism of these LIMs, as shown in Figs. 4 and 5. In the conventional CMOS NAND and NOR gates, the VTC corresponding to *V*_OUT_-*V*_IN_ characteristics of the gate combination is shown when static bias is applied, and the VTC only indicates the logical operation of the NAND and NOR gates. In contrast, our dynamic VTC of the NAND and NOR LIM additionally shows memory operation without any power supply voltage and uses the same scheme as CMOS NAND and NOR gate combination. In other words, when input voltages (*V*_IN1_, *V*_IN2_, *V*_DD_, and *V*_SS_) are applied, the logical operation of the NAND and NOR LIM is conducted, as shown in the truth table of Fig. 1c,d. Additionally, unlike CMOS NAND and NOR logic gates, when all supply voltages are applied at 0 V, the memory operation in which the previous logic state is maintained is performed. This is portrayed graphically using a dynamic VTC.

The detailed VTC operation mechanism of the NAND and NOR LIM is depicted in Fig. 4a,d, which includes the sequence of operation under a pulse value of *V*_IN2_ = 1.0 V. Figure 4b,e show timing diagrams reflecting the dynamic operation of a pulse width of 5 ns for input logic and a hold time of 10 ns for memory operation under all zero-bias conditions. The notation ① indicates the starting point, and the notations ②–⑨ indicate a sequence of operating *V*_IN1_ clockwise. The notation ①, the starting point of the graph, indicates the input logic ‘11’ of the NAND and NOR LIM, and its corresponding results, output logic ‘0’s, are shown in the truth table of Fig. 1c,d. The notation ② indicates the ramping (rising/falling) time of 1 ns for the logic operation, which is the time to reach the next operation, notation ③. Notation ③ shows the hold ‘0’ operations that retains the previous output logic ‘0’ under *V*_DD_, *V*_SS_, *V*_IN1_, and *V*_IN2_ of 0.0 V. Notation ④ indicates the ramping time of 1 ns to proceed to the next operation, notation ⑤. Notation ⑤ shows the input logic ‘01’, resulting in an output logic of ‘1’ in the NAND LIM and ‘0’ in the NOR LIM. To move on to the next operation of holding the output logic ‘1’ of NAND LIM and the output logic ‘0’ of NOR LIM, notation ⑥ has a ramping time of 1 ns. The hold ‘1’ and the hold ‘0’ are maintained, which achieves approximately zero static power consumption, as shown in notation ⑦. After notation ⑧ operates, which comprises a ramping time of 1 ns, the input logic ‘11’ corresponding to notation ⑨ operates. Finally, the closed memory windows of the dynamic VTCs are completed as *V*_IN1_ sweeps clockwise to 1.0 V. Figure 4c,f are summaries of the logic operations performed in Fig. 4a,d, respectively. The memory windows as shown in Fig. 4a,b indicate not only the input logic combinations ‘11’, ‘01’, and ‘11’ of the NAND and NOR LIM but also the memory operation of the output logic under all zero bias conditions. Figure 5a,b show the dynamic NAND and NOR VTCs that *V*_IN1_ operates from 1.0 V in the clockwise direction with different *V*_IN2_ pulses of − 1.0 V, − 0.5 V, 0.0 V, 0.5 V, and 1.0 V, respectively. All starting points are *V*_IN1_ = 1.0 V in the dynamic VTC, which represents the initial logic combinations, and the operating sequence of the dynamic VTCs as shown in Fig. 5 corresponds to the explanation in Fig. 4. Additional analyses of other combinations and the VTC corresponding to the anticlockwise direction sweeping *V*_IN2_ with different pulses of *V*_IN1_ are shown in supplementary sections 4–9.

Consequently, the memory windows of the dynamic VTC have important aspects for interpreting the NAND and NOR LIM operation under the same dynamic bias conditions, as shown in Fig. 2a,b. Note that the sharp transition performance reveals that *p*- and *n*-FBFETs utilized in NAND and NOR LIM have steep switching characteristics of near-zero *SS*. Therefore, our NAND and NOR LIMs can accurately perform not only the output voltage swings for well-defined logic ‘0’ and logic ‘1’ voltages but also memory operation at a range of voltage of the memory windows.

## Conclusion

The presented NAND and NOR LIM composed of two-component SiNW *p*- and *n*-FBFETs exhibit logic operations with a fast processing speed of 5 ns and memory characteristics that retain the output logic states for input logic combination without any additional bias supplies, based on a positive feedback mechanism. The dynamic VTCs verify the electrical characteristics of two-input gates, which correspond to the NAND and NOR LIM operations. This work contributes to a timely discussion of new computing systems, proposing the NAND and NOR LIMs to improve the energy efficiency and latency compared to traditional computing architecture with the von Neumann bottleneck.

## Methods

This simulation was conducted based on the two-dimensional structure of the FBFETs using a TCAD simulator (Synopsys’ Sentaurus™, Version O_2018.06, https://www.synopsys.com/ silicon/tcad.html) to investigate the NAND and NOR LIM^26^. This simulation based on the two-dimensional structure was performed to investigate and understand the overall functionality of the LIMs comprising nanoscale single-gated FBFETs, gaining an insight into its future memory applications; the two-dimensional simulation results can be sufficient to demonstrate the functionality of three-dimensional devices^42,43^. The simulation models for analyzing the FBFETs included Fermi–Dirac carrier statistics, doping-dependent mobility, high-field saturation mobility, Lombardi including a transverse field-dependent model of mobility with phonon scattering and surface roughness scattering according to Matthiessen’s rule, and Slotboom bandgap-narrowing. We also considered the Shockley–Read–Hall recombination and Auger recombination. We used the default parameters for these models using the Sentaurus Device in our simulations. All dynamic operations in this study were performed through transient simulation.

## Supplementary Information

Supplementary Information.
