# Supplementary material for: NAND and NOR logic-in-memory comprising silicon nanowire feedback field-effect transistors
Source: Sci Rep. 2022 Mar 7;12:3643. doi: 10.1038/s41598-022-07368-0 (PMC8901646; doi:10.1038/s41598-022-07368-0)
Supplement: Supplementary file 1 — Supplementary Information. [file 41598_2022_7368_MOESM1_ESM.docx]

Supplementary Information for

**NAND and NOR logic-in-memory comprising silicon nanowire feedback field-effect transistors**

**Yejin Yang^1^, Juhee Jeon^2^, Jaemin Son^2^, Kyoungah Cho ^2^ and Sangsig Kim^1,2*^**

^1^Department of Semiconductor Systems Engineering, Korea University, Republic of Korea

^2^Department of Electrical Engineering, Korea University, 145 Anam-ro, Seongbuk-gu,
Seoul 02841, Republic of Korea

**Table of contents**

Supplementary Section 1 | Cross-sectional view of a single-gated SiNW FBFETs

Supplementary Section 2 | Logic performance of the NAND and NOR LIMs

Supplementary Section 3 | Retention times of holding the output logic state of the NAND and NOR LIM without the parasitic load capacitance.

Supplementary Section 4 | Dynamic voltage-transfer characteristics of the NAND LIM sweeping *V*_IN1_ analyzed using timing diagrams and truth tables.

Supplementary Section 5 | Dynamic voltage-transfer characteristics of the NAND LIM sweeping *V*_IN2_ with different pulse values of *V*_IN1_.

Supplementary Section 6 | Dynamic voltage-transfer characteristics of the NAND LIM sweeping *V*_IN2_ analyzed using timing diagrams and truth tables.

Supplementary Section 7 | Dynamic voltage-transfer characteristics of the NOR LIM in a sweep of *V*_IN1_ analyzed using timing diagrams and truth tables.

Supplementary Section 8 | Dynamic voltage-transfer characteristics of the NOR LIM sweeping *V*_IN2_ with different pulse values of *V*_IN1_.

Supplementary Section 9 | Dynamic voltage-transfer characteristics of the NOR LIM in a sweep of *V*_IN2_ analyzed using timing diagrams and truth tables.

**Supplementary Section 1.** Cross-sectional view of single-gated SiNW FBFETs

Figure S1 shows a cross-sectional view of the *p*- and *n*-FBFETs utilized in the NAND and NOR LIMs. The dimensional parameters of the *p*-FFBET, shown in Fig. S1(a), are a channel length (*L*_CH_) of 150 nm, a gate oxide thickness (*T*_OX_) of 6 nm, *p*^+^ drain and *n*^+^ source region lengths of 50 nm, and a silicon channel thickness (*T*_Si_) of 25 nm. The non-gated channel length (*L*_NG_) and gated channel length (*L*_G_) are 1/2*L*_CH_. All the doping concentrations of the *p*^+^ drain, *n*^+^ source, *L*_NG_, and *L*_G_ regions were 1 × 10^20^ cm^-3^. The dimensional parameters of the *n*-FFBET shown in Fig. S1(b) are an *L*_CH_ of 200 nm, a *T*_OX_ of 3.4 nm, *p*^+^ drain and *n*^+^ source region lengths of 50 nm, and a *T*_Si_ of 15 nm. The *L*_NG_ and *L*_G_ were 1/2*L*_CH_. The doping concentrations were 1 × 10^20^ cm^-3^ for the *p*^+^ drain and *n*^+^ source regions, 5 × 10^19^ cm^-3^ for the *L*_NG_ region, and 9 × 10^19^ cm^-3^ for the *L*_G_ region. The work functions of the gate metals are 4.1 eV for the *p*-FBFET and 5.0 eV for the *n*-FBFET.


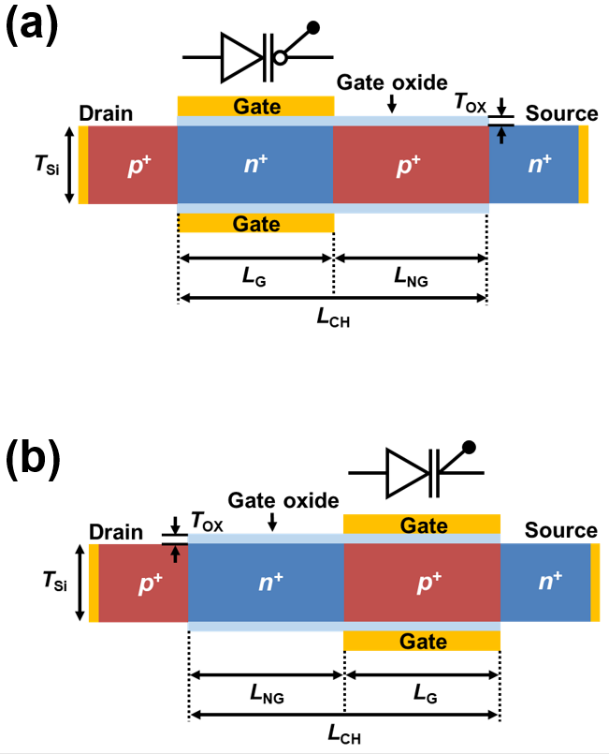


**Figure S1.** Cross-sectional view of single-gated SiNW (a) *p*- and (b) *n*-FBFETs.

**Supplementary Section 2.** Logic performance of the NAND and NOR LIM

Figures S2(a) and (b) show the output voltage corresponding to the logic operation of the NAND and NOR LIM connected to the supply voltages (*V*_DD_ and *V*_SS_). The plots shown in Figs. S2(a) and (b) illustrate *V*_OUT_ versus time, which corresponds to the output logic when two-input voltage pulses are applied according to time with a certain sequence (‘00’, ‘01’, ‘10’, and ‘11’), that is, the dynamic logic circuit illustrates the logic operation of the LIM, which depends on the characteristics of the FBFETs that maintain the charge carriers in the channel region based on a positive feedback mechanism during the applied logic input pulses [1-4]. In the NAND LIM configuration, as shown in Fig. S2(a), when either one or both input pulses are low (input logic ‘0’) at *V*_DD_ = 1.6 V and *V*_SS_ = −2.5 V, the pull-up network (PUN) consisting of two *p*-FBFETs connected in parallel pulls *V*_DD_ up to *V*_OUT_, while the pull-down network (PDN) consisting of two *n*-FBFETs connected in series is cut off. On the other hand, when both input pulses are high (input logic ‘1’), the PUN is cut off because the two *p*-FBFETs are turned off while the PDN pulls *V*_OUT_ down to *V*_SS_ because the two *n*-FBFETs are turned on. In the NOR LIM configuration, as shown in Fig. S2(b), when both input pulses are low at *V*_DD_ = 2.5 V and *V*_SS_ = −1.6 V, the PUN consisting of two *p*-FBFETs connected in series pulls *V*_DD_ up to *V*_OUT_, while the PDN consisting of two *n*-FBFETs connected in parallel is cut off. On the other hand, when either one or both input pulses are high, the PUN is cut off because one or both of the *p*-FBFETs are turned off, while the PDN pulls *V*_OUT_ down to *V*_SS_ because one or both of the *n*-FBFETs are turned on. Although the output voltage levels exhibit a slight difference due to temporary variation in the number of charge carriers in the channel region when input pulses are applied, the output logic states of the NAND and NOR LIM are well-operated for the combination of input logic gates. Accordingly, the NAND and NOR LIM comprising the FBFETs can be compatible with CMOS gates comprising MOSFETs for logic operation. These results allow for an extension of the NAND and NOR LIM operations under dynamic conditions.


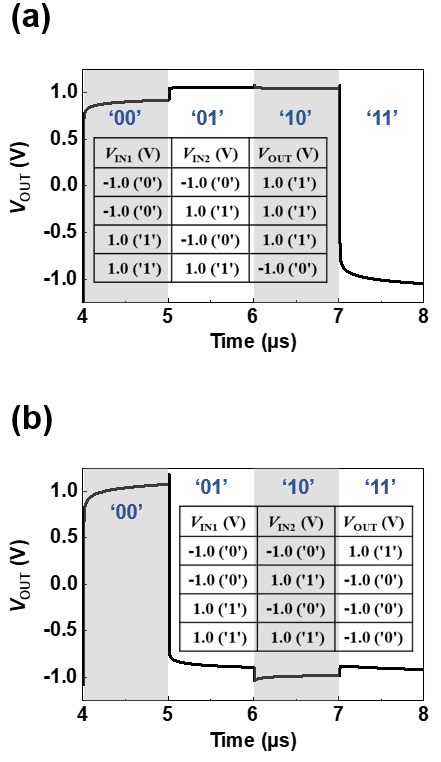


**Figure S2.** Timing diagrams of output voltage of (a) NAND and (b) NOR LIM with supply voltages of *V*_DD_ and *V*_SS_. Insets show truth tables for four logic combinations.

**Supplementary Section 3.** Retention times of holding the output logic state of the NAND and NOR LIM without the parasitic load capacitance.

Figure S3 shows the retention times of holding the output logic state of NAND and NOR LIM without the parasitic load capacitance. To confirm the retention time of the LIMs composed of only FBFETs without external impact, we performed the retention time operation without the parasitic load capacitance. The sequence of the operation is equal in the main manuscript. Subsequently, input voltages corresponding to the input logic with a pulse width of 5 ns were applied, and all zero-bias conditions were maintained for 10 s, as shown in Fig. S3. The retention time is calculated as the time at which the output voltage reaches 37% of the initial *V*_OUT_. As a result, the retention time of the NAND LIM without the parasitic load capacitance corresponding to the input logic sequence ‘00’, ‘01’, ‘10’, and ‘11’ is 1.10 s, 0.66 s, 0.87 s, and >10 s, which represents 37% of the initial *V*_OUT_ as shown in Fig. S3(a). Likewise, the retention time of the NOR LIM without the parasitic load capacitance corresponding to the input logic sequence ‘00’, ‘01’, ‘10’, and ‘11’ is 0.73 s, 5.40 s, 5.66 s, and 7.26 s as shown in Fig. S3(b). As a result, it was confirmed that the presence of the parasitic load capacitor affects retention time. The retention time in the presence of the parasitic load capacitance is longer than when there is no parasitic load capacitance. The retention time that lengthens with the presence of the parasitic load capacitor is the unique performance that appears in the logic circuit composed of the FBFETs. These results show that the FBFET itself can store charge carriers without any external bias although the retention time of each output logic is shorter than when there is the parasitic load capacitance on the output node. Thus, the proposed LIMs composed of FBFETs demonstrate the promising possibility of next-generation memory.


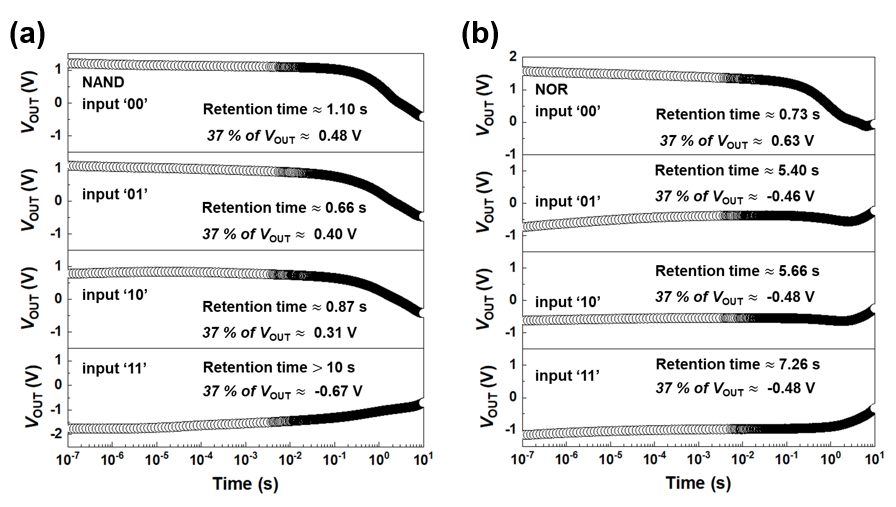


**Figure S3**. **LIM retention characteristics without the parasitic load capacitance.** (a) Time-dependent retention characteristics for *V*_OUT_ of NAND LIM, following the input logic of ‘00’, ‘01’, ‘10’, and ‘11’ with each pulse width of 5 ns, respectively. (b) Time-dependent retention characteristics for *V*_OUT_ of NOR LIM, following the input logic of ‘00’, ‘01’, ‘10’, and ‘11’ with each pulse width of 5 ns, respectively. The retention time for output logic is the time corresponding to 37% of the initial *V*_OUT_.

**Supplementary Section 4.** Dynamic voltage-transfer characteristics of the NAND LIM sweeping *V*_IN1_ analyzed using timing diagram and truth table

Figure S4 shows the electrical characteristics of the NAND LIM, reflecting dynamic LIM operation conditions by changing the input node 1 (*V*_IN1_) logic state with a sequence of ‘1’, ‘0’, and ‘1’ while maintaining the input node 2 (*V*_IN2_) logic stage at ‘0’ and ‘1’, respectively. Figures S3(a) and (d) show the dynamic voltage-transfer characteristics (VTC) curves for operating *V*_IN1_ clockwise with pulses of (a) *V*_IN2_ = 1.0 V and (d) *V*_IN2_ = −1.0 V, corresponding to the blue and black lines, respectively (see Fig. 4(a) in the main text). Notation ① indicates the starting point, and notations ②–⑨ indicate a sequence of operating *V*_IN1_ clockwise, as shown in Figs. S4(a) and (d). To explain the consecutive operations in detail, these symbols are also used in Figs. S4(b), (c), (e), and (f). Figures S4(b) and (e) show timing diagrams reflecting the dynamic operation of a pulse width of 5 ns for input logic and a hold time of 10 ns for memory operation under all zero-bias conditions. Notation ①, the starting point of the graph, indicates the input logic ‘11’ (or ‘10’) in the truth table of the NAND LIM, and its corresponding results, output logic ‘0’ (or ‘1’), are also shown in Fig. S4(c) (and Fig. S4(f)). Notation ② indicates a ramping (rising/falling) time of 1 ns for the logic operation, which is the time to reach the next operation, notation ③. Notation ③ shows the hold ‘0’ (or ‘1’) operation that retains the previous output logic ‘0’ (or ‘1’) under *V*_DD_, *V*_SS_, *V*_IN1_, and *V*_IN2_ of 0.0 V. Notation ④ indicates a ramping time of 1 ns to proceed to the next notation ⑤. Notation ⑤ indicates the input logic ‘01’ (or ‘00’), and both output logics are set to ‘1’. To move to the next operation of holding output logic ‘1’, notation ⑥ indicates a ramping time of 1 ns. The hold ‘1’ values are maintained, which achieves approximately zero static power consumption shown in notation ⑦. After notation ⑧ operates, which represents a ramping time of 1 ns, the input logic ‘11’ (or ‘10’) corresponding to notation ⑨ operates. Finally, the closed memory windows of the dynamic VTCs are completed as the *V*_IN1_ sweeps clockwise to 1.0 V. Figures S4(a) shows a large memory window that indicates not only the input logic combinations ‘11’, ‘01’, and ‘11’ of the NAND LIM, but also the memory operation of the output logic under all zero bias conditions. Figure S4(d) shows a small memory window because the input logic combinations ‘10’, ‘00’, and ‘10’ result in similar output logic values of ‘1’. The notations ①, ⑤, and ⑨ operations, corresponding to the logic operation of the NAND LIM, are summarized in Figs. S4(c) and (f), respectively.


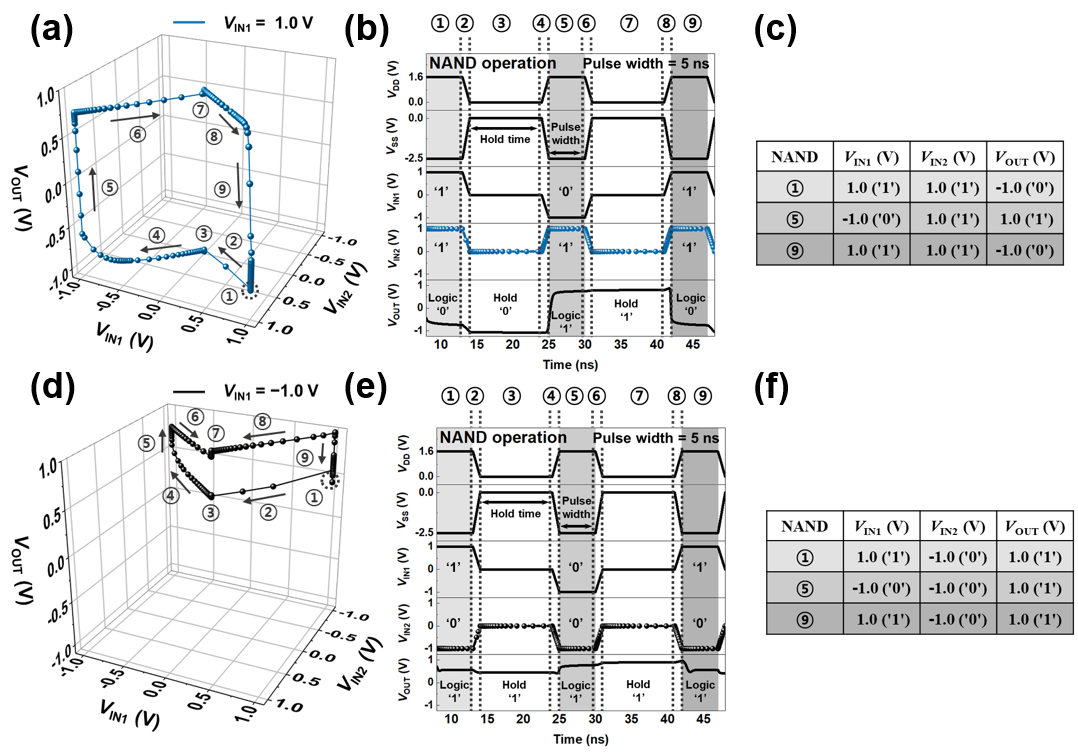


**Figure S4.** Dynamic VTCs of the NAND LIM sweeping *V*_IN1_ with pulse values of (a) *V*_IN2_ = 1.0 V and (d) *V*_IN2_ = −1.0 V. (b) and (e) indicate timing diagrams corresponding to the sequential dynamic VTCs, respectively. Tables (c) and (f) summarize the performed logic operations, respectively.

**Supplementary Section 5.** Dynamic voltage-transfer characteristics of the NAND LIM sweeping *V*_IN2_ with different pulse values of *V*_IN1_.


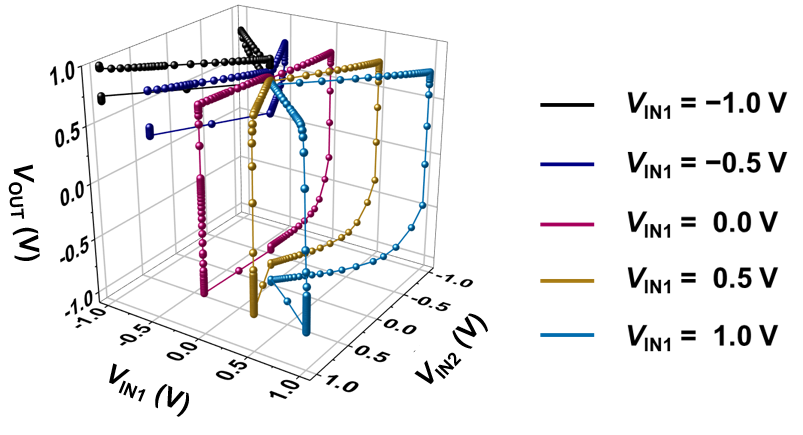


**Figure S5.** Dynamic VTCs of the NAND LIM sweeping *V*_IN2_ with different pulse values of *V*_IN1_.

**Supplementary Section 6.** Dynamic voltage-transfer characteristics of the NAND LIM sweeping *V*_IN2_ analyzed using timing diagrams and truth tables.

To confirm that the same LIM operation is performed regardless of the sweeping input nodes (*V*_IN1_ and *V*_IN2_), the VTCs shown in Fig. S6 are performed by changing the *V*_IN2_ state while maintaining the *V*_IN1_ logic state, as opposed to Fig. S4 in the NAND LIM operation. Figures. S6(a) and (d) show the dynamic VTC curves of operating *V*_IN2_ anticlockwise from 1.0 V while maintaining *V*_IN1_ of (a) 1.0 V and (d) −1.0 V, corresponding to the blue and black lines, respectively (see Fig. S5 in Supplementary Section 5). Notations ①–⑨ indicate a sequence of operating *V*_IN2_ anticlockwise, as shown in Figs. S6(a) and (d). Notation ① indicates the input logic ‘11’ (or ‘01’) in the truth table of the NAND LIM. Notation ② indicates a ramping time of 1 ns, which is the time to reach the next operation, notation ③. Notation ③ indicates hold ‘0’ (or ‘1’) that maintains the previous output logic ‘0’ (or ‘1’) operation under hold bias conditions. Notation ④ represents a ramping time of 1 ns to proceed to notation ⑤. Notation ⑤ shows that both output logic values are set to ‘1’ by applying input logic values of ‘10’ (or ‘00’). Notation ⑥ represents a ramping time of 1 ns to perform the next operation. The hold ‘1’s are maintained under all zero bias conditions shown in notation ⑦. After notation ⑧, a ramping time of 1 ns, notation ⑨ indicates the operation of the input logic ‘11’ (or ‘01’), and thereby the memory windows of the dynamic VTC are completed. Figures S6(c) and (f) are summaries of the logic operations performed in Figs. S6(a) and (d), respectively. Consequently, the memory windows, as shown in Figs. S6(a) and (d), show all the states of the NAND LIM operations under dynamic conditions, as shown in Fig. 2(a) of the main text. Moreover, the operations shown in Figs. S4 and S6 indicate that the same output logic is performed when the NAND LIM operation is performed regardless of the *V*_IN1_ and *V*_IN2_ that sweep.


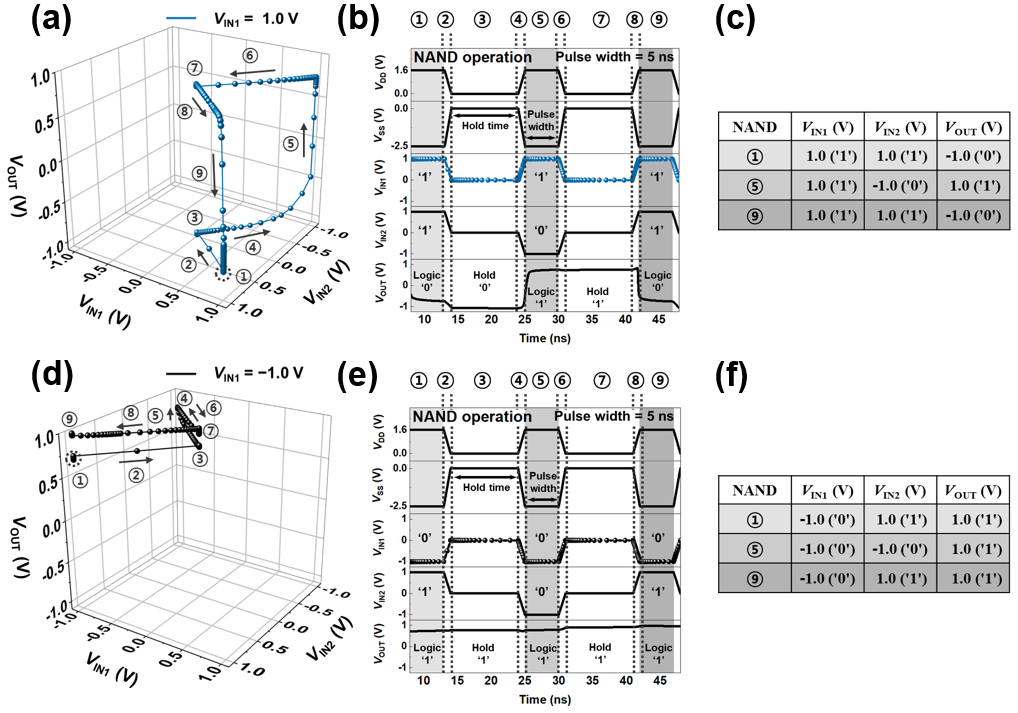


**Figure S6.** Dynamic VTCs of the NAND LIM sweeping *V*_IN2_ with pulse values of (a) *V*_IN1_ = 1.0 V and (d) *V*_IN1_ = −1.0 V. (b) and (e) indicate timing diagrams corresponding to the sequential dynamic VTCs, respectively. Tables (c) and (f) summarize the performed logic operations, respectively.

**Supplementary Section 7.** Dynamic voltage-transfer characteristics of the NOR LIM in a sweep of *V*_IN1_ analyzed using timing diagrams and truth tables.

Figure S6 shows the dynamic VTCs reflecting the NOR LIM operation. Figures S7(a) and (d) show the dynamic VTC curves of operating *V*_IN1_ clockwise from 1.0 V while maintaining (a) *V*_IN2_ of 1.0 V and (d) −1.0 V, corresponding to the blue and black lines, respectively (see Fig. 4(b) in the main text). Notations ①–⑨ indicate a sequence of operating *V*_IN1_ clockwise, as shown in Figs. S7(a) and (d). Notation ① indicates input logic ‘11’ (or ‘10’) in the truth table of the NOR LIM. Notation ② represents a ramping time of 1 ns, which is the time required to reach notation ③. Notation ③ reflects the hold ‘0’ operation that stores the previous output logic ‘0’ under all zero bias conditions, respectively. Notation ④ indicates a ramping time of 1 ns to proceed to notation ⑤. Notation ⑤ indicates that the output logic is set to ‘0’ (or ‘1’) by applying input logic ‘01’ (or ‘00’). Notation ⑥ indicates a ramping time of 1 ns to perform the next operation. The hold ‘0’ (or ‘1’) is maintained under all zero-bias conditions shown in notation ⑦. After notation ⑧, indicating a ramping time of 1 ns, notation ⑨ shows the operation of input logic ‘11’ (or ‘10’), and thereby the memory windows of the dynamic VTC are completed. The memory windows, as shown in Figs. S7(a) and (d) reveal not only the logic operation of the NOR LIM but also the operation that stores the output logic, and the logic operation of the NOR LIM is summarized in Figs. S7(c) and (f), respectively.


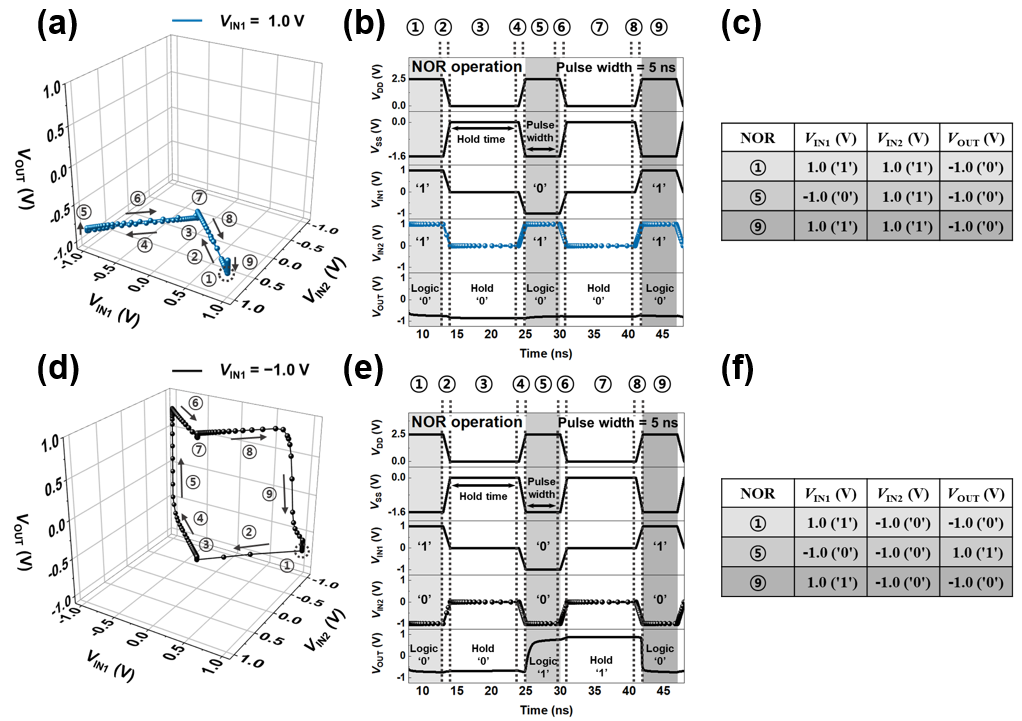


**Figure S7.** Dynamic VTC of the NOR LIM in a sweep of *V*_IN1_ with pulse values of (a) *V*_IN2_ = 1.0 V and (d) *V*_IN2_ = −1.0 V. (b) and (e) indicate timing diagrams corresponding to the sequential dynamic VTCs, respectively. Tables (c) and (f) summarize the performed logic operations, respectively.

**Supplementary Section 8.** Dynamic voltage-transfer characteristics of the NOR LIM in a sweep of *V*_IN1_


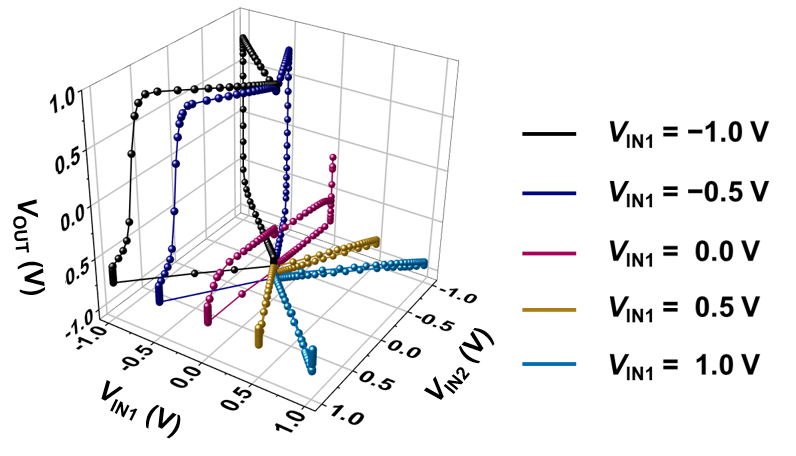


**Figure S8.** Dynamic VTCs of the NOR LIM sweeping *V*_IN2_ with different pulse values of *V*_IN1_.

**Supplementary Section 9.** Dynamic voltage-transfer characteristics of the NOR LIM in a sweep of *V*_IN2_ analyzed using timing diagrams and truth tables.

Figure S8 shows a similarity to the operation shown in Fig. S7, except for changing *V*_IN2_ and maintaining *V*_IN1_ in the NOR LIM operation. Figures S9(a) and (d) show the dynamic VTC curves of operating *V*_IN2_ anticlockwise from 1.0 V while maintaining *V*_IN1_ of (a) 1.0 V and (d) −1.0 V, corresponding to the blue and black lines, respectively (see Fig. S8 in Supplementary Section 8). Notations ①–⑨ indicate a sequence of operating *V*_IN2_ anticlockwise, as shown in Figs. S9(a) and (d). Notation ① indicates the input logic ‘11’ (or ‘01’) in the truth table of the NOR LIM. Notation ② represents a ramping time of 1 ns, which is the time required to reach notation ③. Notation ③ indicates the hold ‘0’ operation that holds the previous output logic ‘0’ under the hold bias conditions. Notation ④ indicates a ramping time of 1 ns to proceed to notation ⑤. Notation ⑤ shows that the output logic is set to ‘0’ (or ‘1’) by applying the input logic ‘10’ (or ‘00’). Notation ⑥ indicates a ramping time of 1 ns to perform the next operation. The hold ‘0’ (or ‘1’) is maintained under all zero-bias conditions shown in notation ⑦. After notation ⑧, indicating a ramping time of 1 ns, the input logic ‘11’ (or ‘01’) operates as shown in notation ⑨, and thereby the memory windows of the dynamic VTC are completed. Figures S9(c) and (f) are summaries of the logic operations conducted in Figs. S9(a) and (d), respectively. The NOR LIM operation is a well-defined logical output regardless of the *V*_IN1_ and *V*_IN2_ that sweep, as shown in Figs. S7 and S9. As a result, the proposed dynamic VTCs provide new evidence for the proposed LIM operation interpretation under dynamic conditions.


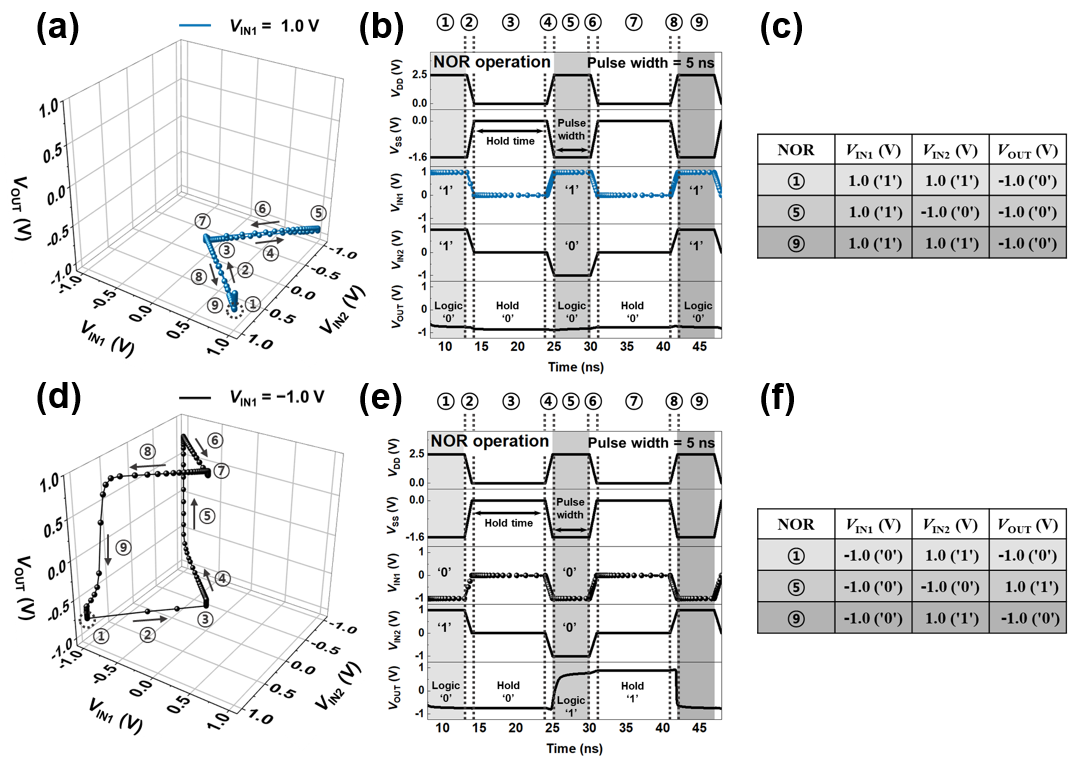


**Figure S9.** Dynamic VTC of the NOR LIM in a sweep of *V*_IN2_ with pulse values of (a) *V*_IN1_ = 1.0 V and (d) *V*_IN1_ = −1.0 V. (b) and (e) indicate timing diagrams corresponding to the sequential dynamic VTCs, respectively. Tables (c) and (f) summarize the performed logic operations, respectively.

**References:**

1. Kim, J.-S. *et al*. Dynamic Logic Circuits Using a-IGZO TFTs. *IEEE Trans. Electron Devices* **64**, 4123-4130 (2017).

2. Kim, M. *et al*. Steep switching characteristics of single-gated feedback field-effect transistors. *Nanotechnology* **28**, 055205 (2017).

3. Kim, Y. *et al*. Switchable‐Memory Operation of Silicon Nanowire Transistor. *Adv. Electron. Mater.* **4**, 1800429 (2018).

4. Woo, S. *et al*. Device design of single-gated feedback field-effect transistors to achieve latch-up behaviors with high current gains. *Curr. Appl. Phys.* **20**, 1156-1162 (2020).
